# Spinal Cord Ischemia in Open and Endovascular Aortic Repair

**DOI:** 10.1055/s-0042-1756669

**Published:** 2022-12-15

**Authors:** Enrico Rinaldi, Diletta Loschi, Nicola Favia, Annarita Santoro, Roberto Chiesa, Germano Melissano

**Affiliations:** 1 Division of Vascular Surgery, IRCCS San Raffaele Scientific Institute, Vita-Salute San Raffaele University, Milan, Italy

**Keywords:** spinal cord ischemia, neurological deficits, open repair, endovascular repair

## Abstract

Despite the improvements, spinal cord ischemia is still one of the major and most dramatic potential complications after thoracic and thoracoabdominal aortic treatments, for both open and endovascular procedures. A multimodal approach, which includes several intraoperative and postoperative maneuvers, may contribute to optimizing the spinal cord tolerance to ischemia. The aim of this article is to report the different techniques employed to improve spinal cord perfusion, directly and indirectly through collateral circulation.

## Introduction

Spinal cord ischemia (SCI) is one of the major and most dreadful complications that may follow thoracic and thoracoabdominal (TAA) aortic repair, both open and endovascular, and it may result in devastating physical disabilities, but also in a much-reduced survival at follow-up.

SCI may cause several degrees of neurological deficits from temporary or permanent paraparesis to complete flaccid paraplegia. However, mobility impairment is only a part of the clinical syndrome. The lack of mobility and sensitivity in the lower half of the body is responsible for bedsores that can evolve into severe infections. Fecal and urinary incontinence may also result in recurrent infections and are psychologically poorly tolerated. Deep venous thrombosis is another possible adjunctive complication in patients with complete or partial lack of mobility. Moreover, postoperative SCI may affect elderly patients, with preexisting respiratory, cardiac, and renal comorbidities. This explains the very poor survival rate of patients with the more severe forms.

The pathogenesis of spinal cord (SC) damage during TAA procedures, while multifactorial, is mainly due to an ischemic insult. Concerning the onset of symptoms, neurological damage may be immediate (during or at the end of the procedure) or delayed (after a period of normal neurological function). Immediate SCI mainly results from a temporary or permanent reduction of SC blood supply. A delayed deficit may be due to an SC perfusion impairment but also can result from an ischemia/reperfusion mechanism, with SC swelling edema within the bony spinal canal and a consequent increase in cerebrospinal fluid (CSF) pressure. Moreover, some authors speculate that the intraoperative ischemic insult induces a programmed neuronal cell death. 1 Others, also consider the role of late thrombosis of intercostal arteries as another possible pathogenetic factor.

During recent decades, improvements have been made in both open and endovascular TAA repairs. However, SCI is still an open issue in this field, and its pathophysiology is still poorly understood. A multimodal approach, based on a deep knowledge of the SC anatomy, along with different protective adjuncts, may be considered to prevent SCI for both open and endovascular TAA repairs.

## Spinal Cord Anatomy

SC blood supply is characterized by extreme interindividual variability, but it generally presents two vasculature pathways, an extrinsic and an intrinsic one.

The extrinsic system includes:

- The longitudinal arterial trunks:
  - Anterior spinal artery (single).
  - Posterior or posterolateral spinal arteries (double, rarely single).
- The pial plexus or perimedullary vascular network.

The intrinsic system includes two different systems:

- a central system (centrifugal) fed by the sulcal arteries.
- a peripheral system (centripetal) with perforating branches originating from the pial network.
  The intercostal arteries divide three times to reach the anterior spinal artery (ASA) which supplies blood to the spinal gray matter:
- The first branch of the intercostal artery is the nervomedullary artery.
- The latter divides into an anterior and posterior radicular artery.
- The anterior radicular artery divides into a descending and an ascending branch.

**Fig. 1:**
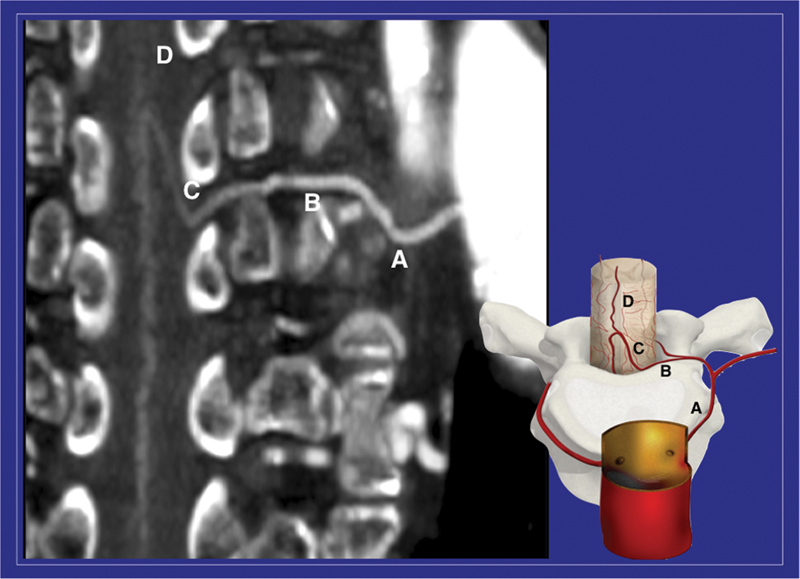
With preoperative computed tomography angiography, using postprocessing tools, the whole path of the arterial feeder to the spinal cord can be visualized, from the aorta to the anterior spinal artery (A: intercostal artery; B: anterior radicular artery; C: arteria radicularis magna or artery of Adamkiewicz; D: anterior spinal artery).

An anastomotic channel between ascending and descending branches of neighboring anterior radicular arteries creates the ASA during the embryonic and fetal stages. However, in the adult, only at few levels, the anterior and posterior radicular arteries cross the dura to reach the surface of the medulla. In the thoracolumbar region, one (occasionally two or three) anterior radicular artery is dominant in caliber and is therefore called the great radicular artery or arteria radicularis magna (ARM), or the artery of Adamkiewicz ( Fig. 1 ). A more detailed description of SC vascularization anatomy has been previously published. 2

Many other vessels provide an inflow to these systems, such as the subclavian artery and the hypogastric arteries. Griepp and Griepp 3 introduced the “collateral network concept,” detailing the redundancies in the blood supply to the SC and its significant anatomic variability. While this “collateral network” may guarantee adequate vascularization in many instances, this is not always the case in an acute setting. Aortic interventions could affect at different levels the inflow arteries of the network, and this may explain the physiopathology of SC ischemia in many circumstances. Deep knowledge of the SC vasculature anatomy in the individual patient is therefore essential for accurate risk stratification and a tailored approach focused on SCI prevention.

## Mechanism of Spinal Cord Injury and Prevention Strategies during Thoracoabdominal Open Repair

A temporary reduced perfusion of the SC feeders is substantially inevitable during aortic cross-clamping, and the sacrifice of some intercostal/lumbar arteries is often needed surgically. Several prevention protocols and treatment strategies have been proposed over recent years to maintain an adequate SC perfusion during open repair.

### Minimize Spinal Cord Ischemic Time

**Fig. 2:**
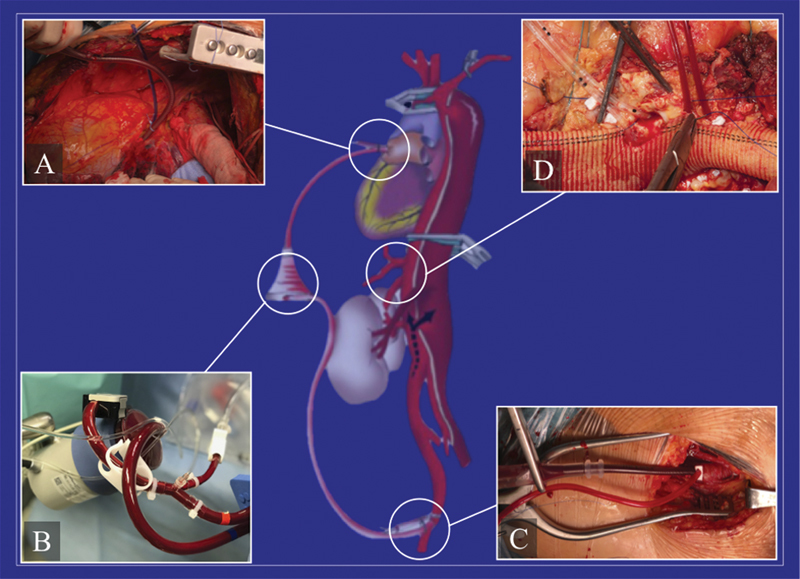
Schematic view of left heart bypass. ( **A** ) A 20-Fr cannula is inserted in one of the left pulmonary veins for the arterial blood drainage. ( **B** ) Through a centrifugal pump, the oxygenated blood is routed into the left femoral artery for synchronous proximal (visceral and intercostal vessels) and ( **C** ) distal perfusion during sequential clamping, using a nonocclusive femoral cannula. ( **D** ) A “Y” connector provides two occlusion/perfusion catheters for selective visceral perfusion with blood.

During TAA open surgery, the aortic cross-clamp time is one of the most significant predictors of postoperative SCI, with a reported incidence up to 27% in patients with an aortic cross-clamp time >60 minutes. Thus, an expeditious aortic surgery has generally been advocated since the early years of this surgery. In addition, sequential aortic clamping and techniques for distal aortic perfusion were introduced to maintain the blood supply of the SC feeding vessels during the aortic clamping time. Left heart bypass (LHBP) associated with sequential clamping, compared with the simple “clamp and sew” technique, has been demonstrated to be protective against SCI. 4 LHBP plays a crucial role, especially in case of extensive repair and when unexpected complications occur, and its effectiveness in reducing the SCI rate has been recently confirmed by experienced aortic centers ( Fig. 2 ). 5

### Preserve Spinal Cord Blood Supply

**Fig. 3:**
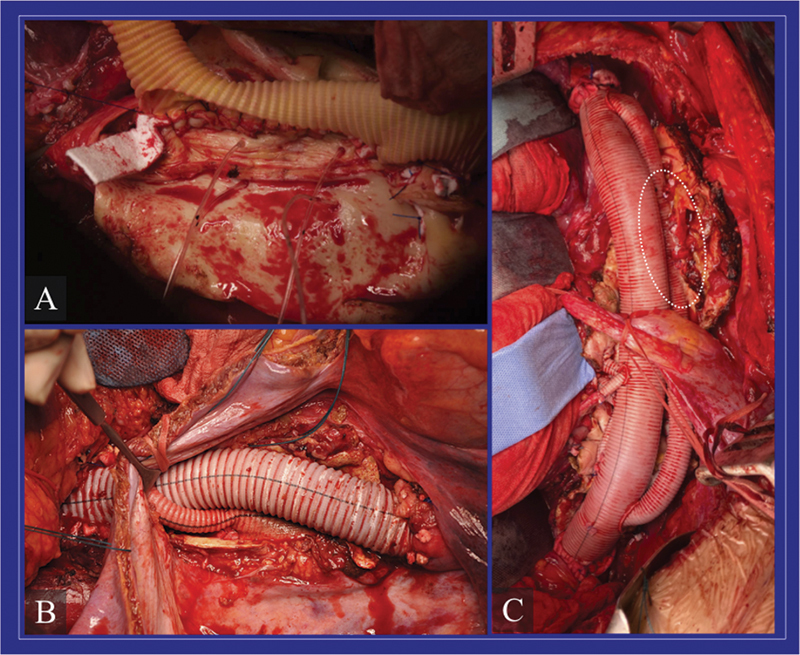
Critical intercostal arteries reattachment during thoracoabdominal aortic aneurysm open surgical repair with three different techniques. ( **A** ) An aortic island including the origin of several intercostal arteries is reattached to a fenestration created on the aortic graft. ( **B** ) Intercostal arteries are reattached selectively to the graft via 6/8-mm interposition grafts. ( **C** ) Another possible way to reattach critical intercostal arteries is represented by the “loop graft”; a 14/16-mm is anastomosed proximally and distally to the aortic graft. A fenestration is created in this loop graft to reattach the origin of multiple intercostal arteries (dotted circle). TAAA, thoracoabdominal aortic aneurysm.

During TAA open repair, it is possible to reattach some intercostal arteries, but the effective role of reimplantation in terms of SCI prevention remains controversial. Some authors advocate an expeditious aortic repair with no intercostal artery reattachment, being confident in the SC collateral network. 6 However, the protective role of critical intercostal artery (T8–L2) reattachment to reduce the risk of postoperative SCI has been extensively demonstrated over the years. 7 With this approach, during the procedure, patent intercostal critical arteries are temporarily occluded to prevent back-bleeding and then selectively reattached to the graft by means of an aortic patch or graft interposition ( Fig. 3 ). This procedure, however, is time-consuming, and large aortic patches may be prone to future dilatation. Thus, it would probably be better to avoid unnecessary reattachments, especially in patients with connective tissue disorders and, in general, in patients with bad quality aortic tissue.

Recent advances in preoperative imaging may play a role in planning selective reimplantation of critical intercostal feeders. Modifications of intraprocedural neurophysiologic monitoring tools are also extremely helpful to identify critical SC feeders.

### Increased Spinal Cord Tolerance to Ischemia

A neuronal injury may develop rapidly after ischemia under normothermic conditions.

Mild systemic hypothermia (32–34°C) may play a protective role on SC, decreasing SC metabolic demands, with consequent attenuation of the inflammatory cascade response. Some authors perform extensive TAA repair under deep hypothermia (15–18°C) and circulatory arrest, gaining the maximum advantage from the protective effects of hypothermia. 1 However, this technique may be limited by coagulopathy and pulmonary and cerebral complications. A selective SC hypothermic protection, by regional cooling with an infusion of cold 4°C saline solution in the epidural space during ischemic periods, has also been proposed, with interesting results. 8

### Optimization of Spinal Cord Perfusion

Optimizing SC perfusion by raising arterial systemic blood pressure and reducing CSF pressure are also key points for the prevention and treatment of SCI.

#### Arterial Systemic Blood Pressure

Hemodynamic stability during the procedure and the postoperative period is very important, and, in general, the mean arterial pressure (MAP) should be maintained over 70 mm Hg. The surgeon plays a major role to ensure the control of hemorrhage, and anesthesiologist and perfusionist need to be prepared to manage large blood losses and to ensure continuous organ perfusion.

Equally, an intensive care unit needs to be familiar with all aspects of TAA postoperative care to maintain adequate hemodynamic stability in the postoperative period. Arterial pressure should be monitored carefully after open TAA repair to avoid unintentional postoperative hypotension that may precipitate SCI.

Extensive TAA repair is a demanding procedure for the whole cardiovascular system, and many of these patients come to surgery with preoperative coronary lesions. To avoid the hemodynamic instability secondary to perioperative cardiac events, an accurate preoperative cardiac evaluation is advocated in all patients undergoing elective TAA repair. 9

#### Cerebrospinal Fluid Pressure

**Fig. 4:**
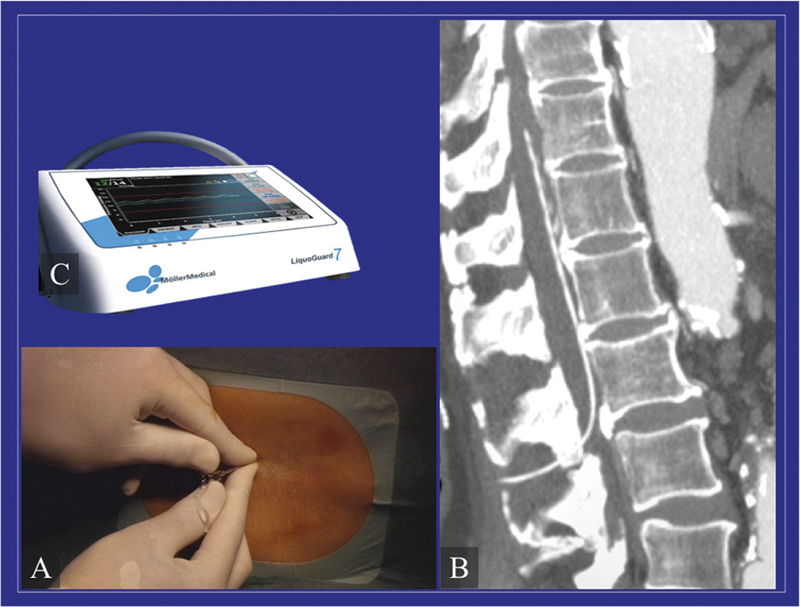
( **A** ) Once the dura has been punctured with the introducer needle, ( **B** ) a drainage catheter is inserted 8 to 10 cm along the intradural space. The catheter is then connected to a pressure transducer, and the fluid is drained to keep the pressure below 10 cm H _2_ O. ( **C** ) Automated systems, such as Liquoguard, are available for this purpose.

CSF pressure rises immediately after SC perfusion impairment, and this mechanism, coupled with decreased SC perfusion pressure, may be one of the major causes of SCI. The preoperative placement of SC drainage allows intra- and postoperative CSF continuous pressure monitoring and drainage and represents a widely practiced technique during TAA surgery. CSF drainage to maintain CSF pressure <10 cm H _2_ 0 has been demonstrated to be effective in paraplegia reduction in cases of extensive TAA repair and also in cases of delayed onset of paraplegia. 10 Although the safety of CSF drainage appears to be acceptable, serious and even fatal complications associated with its placement have been reported. 11 Nowadays, CSF drainage may be performed with an automated device with several advantages that have been previously reported ( Fig. 4 ). 12

### Early Detection of Spinal Cord Ischemia

Early detection of SCI is critical to allow prompt intervention before ischemia evolves to infarction and consequent permanent neurological deficits. In patients under general anesthesia, neurological monitoring of SC function can be obtained with somatosensory-evoked potentials (SSEP), and motor evoked potentials (MEP), or both. Technical details of these techniques have previously been extensively reported. 13 The immediate detection of intraoperative SCI, obtained with the usage of MEP and SSEP monitoring, allows one to trigger prompt anesthesiologic and surgical maneuvers for maximizing SC perfusion and potentially rapidly reversing SC injury.

In patients with MEP and SSEP modifications, increasing distal aortic pressure with LHBP, and CSF drainage are possible ways to restore an adequate SC perfusion. Moreover, aortic replacement performed with sequential cross-clamping maximizes the effect of distal perfusion and may be helpful to identify the critical segments of the aorta that supply to the SC, with possible intercostal artery reattachment. Notably, SC neurological monitoring can be affected by anesthetic agents, which may induce a depressed neural response; thus, sometimes false positive results may be obtained.

Other noninvasive methods to obtain valuable monitoring of SC perfusion have been proposed. In particular, with near-infrared spectroscopy, an effective monitoring of paraspinous muscle oxygenation can be obtained, as a surrogate for SC perfusion. 14 An alteration in CSF biochemical markers consequent to SCI can be identified to detect ischemia promptly. However, the usage of these markers in clinical practice is still limited.

## Mechanism of Spinal Cord Injury and Prevention Strategies during Endovascular Repair

When feasible, thoracic endovascular aortic repair (TEVAR) reduces the morbidity of surgical access and avoids the need for aortic cross-clamping. In the last two decades, technological improvements have offered the opportunity to treat even extensive TAA with endovascular procedures, utilizing fenestrated and branched endovascular devices (F/B-EVAR). These procedures incur also an intrinsic risk of SCI, with different pathophysiological patterns from open repair:

- Aortic cross-clamping is avoided
- The number of intercostal arteries sacrificed is larger, and they cannot be reattached to the graft. If compared with open repair, longer aortic segments are covered by the stent graft (SG) to anchor in healthy aortic necks.
- Manipulation of endovascular devices in the diseased aorta may induce embolic complications contributing to the SC.

Endovascular procedures, without the background noise of aortic cross-clamping and intercostal artery reattachment, offer the opportunity to evaluate the physiopathology of SC ischemia and to understand the importance of the different SC feeders without possible confounding factors. An analysis of data collected in the European Registry of Endovascular Aortic Repair Complications pointed out the importance of the various SC feeders. Czerny et al 15 demonstrated that extensive coverage of intercostal arteries by a TEVAR alone is not associated with SC ischemia, while simultaneous closure of at least two vascular territories (left subclavian artery (LSA), intercostal, lumbar, and hypogastric arteries) supplying the SC is relevant, especially if associated with prolonged intraoperative hypotension. Thus, different prevention protocols have been proposed to preserve SC feeders and maintain an adequate SC perfusion, during the endovascular repair.

### Left Subclavian Artery Revascularization

In the recent past, to obtain an adequate proximal aortic neck during TEVAR, an intentional LSA overstenting without LSA revascularization has frequently been performed. However, multiple studies demonstrated that increased risks of stroke and SCI are associated with LSA occlusion without previous revascularization. 16 Nowadays, in case of elective extensive aortic coverage, preventive LSA revascularization is recommended by society guidelines (grade IIa, level C). 9

### Intercostal and Lumbar Arteries Sacrifice

An intrinsic drawback of TEVAR is the sacrifice of the intercostal arteries that arise not only from the aneurysm but also from the healthy aorta at the level of proximal and distal landing zones. The coverage of an aortic segment > 20 cm with TEVAR was reported to be associated with SCI, and recently published experience of extensive TAA repair with F/B-EVAR has also demonstrated a significant association between SCI and the extent of aortic coverage. 17

Different considerations should be made for TEVAR alone and F/B-EVAR procedures. While endovascular repair with F/B-EVAR needs a distal landing zone in the infrarenal aorta in the majority of cases, in cases of pure thoracic endovascular repair, the distal landing zone is generally above the origin of the celiac trunk. During TEVAR, the length of the uncovered distal thoracic aorta and the distance between the SG and the celiac trunk are associated with the risk of SCI, with a risk reduction of 40% for each 2 cm of the distal thoracic aorta above the celiac trunk left uncovered. 18

Thus, accurate preoperative planning and the identification of a distal landing zone able to preserve critical SC feeders, as well as avoiding unnecessary aortic coverage, are key points in SCI prevention during TEVAR.

An anecdotal experimental study with side small caliber branches for intercostal artery preservations during TEVAR has been reported but mid- and long-term results of these pioneering procedures are still unknown.

During extensive TAA endovascular repair with F/B-EVAR, off-the-shelf devices often require more extensive proximal aortic coverage due to intrinsic manufacturing characteristics. 19 While it is possible to avoid the unnecessary sacrifice of the native healthy aorta with custom-made devices, the possibility to preserve ARM feeders during these procedures is limited, and different approaches have been developed.

One interesting concept that has emerged in the last few years is that the SC vasculature may have the potential to be “preconditioned” to better tolerate the occlusion of the segmental suppliers, such as the intercostal or lumbar arteries. This preconditioning may be obtained by staging the aortic procedures so that the occlusion of the segmental feeders is done in two or three steps. This staged approach has been demonstrated to lower the risk of perioperative SCI significantly. 20

**Fig. 5:**
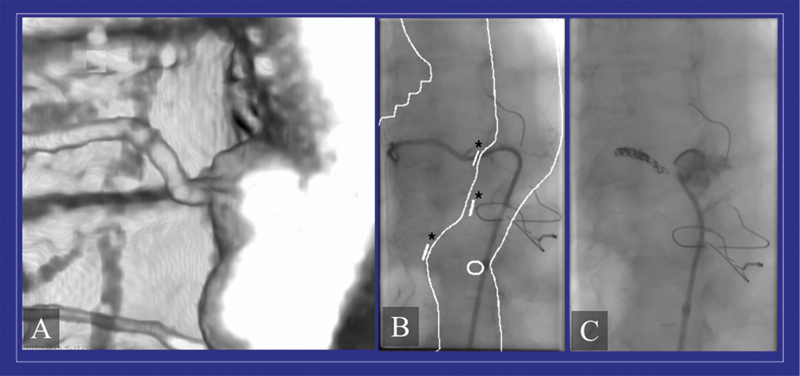
( **A** ) Preoperative computed tomography angiography (AngioCT) is used for the identification of critical intercostal arteries. According to the preoperative spinal cord (SC) vasculature imaging, the occlusion of SC feeders may be planned to induce a collateral network preconditioning. ( **B** ) With the aid of intraoperative adjuncts, such as the fusion technique, an easier identification of the intercostal arteries is possible during the endovascular procedure. With this imaging tool, the preoperative AngioCT is matched with the intraoperative angiography, with the possibility to underline the aortic contour and preoperatively selected vessels, such as the intercostal arteries (*). This approach allows an easier identification of the intercostal vessels for catheterization. ( **C** ) Embolization with coils may be performed to selectively occlude the intercostal artery.

Etz et al 21 proposed selective segmental intercostal artery endovascular coil embolization for preconditioning the collateral network toward minimizing ischemic SC injury, with promising results ( Fig. 5 ). To evaluate the safety and efficacy of this procedure, for both open surgical and endovascular TAA procedures, a prospective, multicenter, international, randomized clinical trial (PAPAartis) has been designed. The study will require several more years to complete enrollment and analyze the results.

### Role of Hypogastric Arteries

The role of the hypogastric arteries as SC feeders has been progressively clarified during the last decade. It has been demonstrated that the chance of developing SCI is significantly higher in patients with an occluded or excluded hypogastric artery. 22 Similarly, a significant correlation between embolic occlusion of the hypogastric arteries and the development of SCI was documented in patients with the endovascular abdominal aortic repair. Maurel et al 23 demonstrated that early restoration of arterial flow to the pelvis and lower limbs significantly reduces the risk of SC ischemia due to the occlusive effect of large sheaths left in place for extended times during complex endovascular procedures to treat thoracoabdominal aneurysms. For these reasons, different surgical and endovascular maneuvers able to restore early antegrade perfusion of hypogastric arteries during endovascular procedures have been proposed.

### Bleeding and Hypotension

Hypotension is an established factor in the genesis of SCI due to a temporary impairment of SC vascularization. After the coverage of intercostal and lumbar arteries, the compensation of the collateral network may be insufficient in case of hemodynamic instability with hypotension, with consequent temporary SC ischemia. Via prompt identification and reversal of hypotension, possible SC deficits may be completely resolved, while prolonged impairment in SC perfusion may lead to irreversible neurological deficits.

During the procedure, excessive blood and fluid loss, caused by arterial injuries or even by surgical procedures like an iliac conduit, may lead to hypotension and have been implicated as a possible cause of SCI during the endovascular repair. 24 Notably, excessive bleeding from endovascular devices may also complicate very long procedures (>300 minutes), even with a percutaneous approach; thus, continuous intraoperative monitoring of the hematocrit is recommended. If a lowering of the systemic pressure is needed during the SG deployment, to avoid a prolonged hypotensive phase, maneuvers such as rapid cardiac pacing and balloon inflation in the inferior vena cava are more rapidly reversible compared with antihypertensive drugs.

During the postoperative course, the use of excessive antihypertensive therapies should be avoided and hemodynamic stability with a MAP >70 mm Hg should be maintained.

### Adjunctive Maneuvers to Detect, Prevent, and Treat Spinal Cord Ischemia

While the protective role of CSF drainage (CSFD) during thoracoabdominal aortic aneurysm (TAAA) open surgical repair has been widely reported, its effective role during endovascular procedures still needs to be completely established. Moreover, CFSD involves a nonnegligible risk of related complications, from minor issues such as headaches to potentially devastating issues such as spinal hematoma or intracranial hemorrhage. For these reasons, the prophylactic use of preoperative CSFD before complex endovascular procedures should be weighed carefully, and many authors suggest its usage only in patients preoperatively considered at high risk for SCI. 11

As for open TAAA repair, intraoperative neuromonitoring with MEP and SSEP may play a role also during complex endovascular procedures for early detection of impairments in SC supply, application of adjunctive maneuvers, and possible prevention of ischemic damage. In contrast to open surgical repair, intercostal artery reattachment is not feasible during endovascular procedures, but other adjunctive maneuvers may increase direct or indirect perfusion of the SC collateral network. Adjunctive maneuvers in response to MEP and SSEP deficits may include incremental changes in mean arterial pressure, as well as an increase in CSFD when a prophylactic spinal drain catheter has been preoperatively placed. If the above-mentioned maneuvers are not sufficient to obtain MEP and SSEP normalization, other technical modifications of the procedure are possible, such as the early restoration of flow to the lower extremity and to the pelvis, or even the interruption of the endovascular repair to restore direct perfusion of the aneurysm sac. 25

### Endoelak Resolution

After endovascular procedures, SCI can be immediate or delayed. While immediate neurological deficits are explained by the occlusion of critical intercostal arteries, delayed SCI may be consequent to hypotension, anemia, or late loss of collaterals. The thrombosis within the excluded aneurysm sac may not be immediate, especially in the case of endoleaks. After endoleak resolution, complete thrombosis of the aneurysmal sac generally occurs, with possible late occlusion of patent critical intercostal arteries, making the collateral network insufficient and incurring delayed onset of SCI. Thus, in the case of SCI, ancillary endovascular maneuvers aimed at voluntarily creating a temporary endoleak have been described.

## Conclusion

Several improvements have been made during recent decades in understanding the mechanisms of SC injury. Nowadays, it is clear that many different factors contribute to cause postoperative paraparesis or paraplegia after both open and endovascular management of TAAA. Side by side with a better knowledge of the different pathophysiological patterns, a multimodal approach to prevent SCI has been developed. A combination of many intraoperative and postoperative maneuvers, both surgical and anesthesiological, may contribute to optimize the SC blood supply and ameliorate its tolerance to ischemia.

With this multimodal approach, better results have been reached compared with the past, but the problem of this dramatic complication is still not solved after either open or endovascular TAAA repair. Further focused research is still needed to improve outcomes.
